# A comprehensive database of amphibian heat tolerance

**DOI:** 10.1038/s41597-022-01704-9

**Published:** 2022-10-04

**Authors:** Patrice Pottier, Hsien-Yung Lin, Rachel R. Y. Oh, Pietro Pollo, A. Nayelli Rivera-Villanueva, José O. Valdebenito, Yefeng Yang, Tatsuya Amano, Samantha Burke, Szymon M. Drobniak, Shinichi Nakagawa

**Affiliations:** 1 Evolution & Ecology Research Centre, School of Biological, Earth and Environmental Sciences, The University of New South Wales, Sydney, New South Wales Australia; 2 Institute of Environmental and Interdisciplinary Science, Department of Biology, Carleton University, Ottawa, Ontario Canada; 3 German Centre for Integrative Biodiversity Research, Halle-Jena-Leipzig, Leipzig, Germany; 4 Helmholtz Centre for Environmental Research (UFZ), Leipzig, Germany; 5 Centre for Biodiversity and Conservation Science, The University of Queensland, Brisbane, Queensland Australia; 6 Centro Interdisciplinario de Investigación para el Desarrollo Integral Regional Unidad Durango (CIIDIR), Instituto Politécnico Nacional, Durango, México; 7 Laboratorio de Biología de la Conservación y Desarrollo Sustentable de la Facultad de Ciencias Biológicas, Universidad Autónoma de Nuevo León, Monterrey, México; 8 Department of Zoology and Human Biology, University of Debrecen, Debrecen, Hungary; 9 Bird Ecology Lab, Instituto de Ciencias Marinas y Limnológicas, Universidad Austral de Chile, Valdivia, Chile; 10 Department of Biosystems Engineering, Zhejiang University, Hangzhou, 310058 China; 11 Department of Infectious Diseases and Public Health, Jockey Club College of Veterinary Medicine and Life Sciences, City University of Hong Kong, Hong Kong, China; 12 School of Biological Sciences, The University of Queensland, Brisbane, Queensland Australia; 13 Institute of Environmental Sciences, Jagiellonian University, Kraków, Poland

**Keywords:** Animal physiology, Climate-change ecology, Ecophysiology, Ecology, Ecology

## Abstract

Rising temperatures represent a significant threat to the survival of ectothermic animals. As such, upper thermal limits represent an important trait to assess the vulnerability of ectotherms to changing temperatures. For instance, one may use upper thermal limits to estimate current and future thermal safety margins (i.e., the proximity of upper thermal limits to experienced temperatures), use this trait together with other physiological traits in species distribution models, or investigate the plasticity and evolvability of these limits for buffering the impacts of changing temperatures. While datasets on thermal tolerance limits have been previously compiled, they sometimes report single estimates for a given species, do not present measures of data dispersion, and are biased towards certain parts of the globe. To overcome these limitations, we systematically searched the literature in seven languages to produce the most comprehensive dataset to date on amphibian upper thermal limits, spanning 3,095 estimates across 616 species. This resource will represent a useful tool to evaluate the vulnerability of amphibians, and ectotherms more generally, to changing temperatures.

## Abstract (summary)

**Table:** 

Measurement(s)	CTmax • Critical thermal maximum • LT50 • Median lethal temperature • Thermal tolerance • Thermal limits
Technology Type(s)	experimental
Factor Type(s)	Location • Conservation status • Environmental temperature • Laboratory temperatures • Body size • Ontogeny • Methodological variation
Sample Characteristic - Organism	Amphibians • Caudata • Amphibia • Frogs • Salamanders • Newts
Sample Characteristic - Environment	natural environment • laboratory environment
Sample Characteristic - Location	Global

## Background & Summary

The Earth is warming at an unprecedented rate, with extreme heat waves being predicted to increase in both frequency and intensity^1^. Understanding how biodiversity will respond to drastic changes in temperatures is thus paramount for their continued survival. One possibility would be to use the thermal tolerance of species to assess changes in species geographic ranges and extinction risk^2,3^. While the extent to which animals tolerate temperatures can be assessed in various ways, the estimation of upper thermal limits—the temperature at which animals lose chances of survival^4–6^—forms a crucial piece of information to project the impacts of ongoing climate change. Notably, experimentally derived upper thermal limits are great proxies of the fundamental thermal niche of animals^7^ and have enabled important findings in ecophysiology. For instance, large empirical studies and syntheses have demonstrated that most ectotherms are intolerant to extreme temperatures^8^, that species inhabiting tropical and marine environments are particularly vulnerable^2,9,10^, that early-life stages represent the weakest link in the life cycle^11–14^, and that the plasticity^14–18^ and evolutionary potential of upper thermal limits is insufficient to compensate for rising temperatures^19–23^.

While previous data compilations were rigorous^2,15–18,24–26^, there are four major areas for improvement to increase their usability and reliability. First, data compilations require a representative and comprehensive sample of the literature to best inform macrophysiological projections. For example, data imputation procedures assume specific missing data patterns (e.g., data missing at random^27^). Thus, any biases in the data may compromise the validity of imputation procedures^28^. While compiling a comprehensive sample of the literature is challenging, one can reach comprehensiveness by systematically reviewing both grey and traditional academic literature from numerous databases^29^. Second, the geographical coverage of most physiological databases is relatively poor^24,30,31^. One way to minimise this bias is to conduct the literature search in non-English languages because a non-negligible amount of non-English-language literature still exists in countries where English is not widely spoken^30,32^. Third, some datasets on thermal tolerance have limited their scope to one comparable estimate per species^24–26^. However, quantifying within-species variation is of particular interest to comparative physiologists^33,34^. In fact, accumulating evidence support that thermal tolerance varies substantially within the same species due to plasticity and local adaptation e.g.^11,14,17,35,36^^,^. Notably, ectotherms raised at higher temperatures tend to tolerate higher temperatures^14–18^. The incorporation of plastic responses, among other forms of intraspecific variation, is thus incredibly important to accurately inform the future of animals on this planet. Fourth, accounting for measures of statistical dispersion (e.g., standard deviation) is vital to weight findings based on their precision because thermal limit estimates are often underpowered^34,37,38^. Performing unweighted analyses puts an unnecessarily large emphasis on low-powered estimates. Moreover, the unavailability of measures of dispersion in datasets constrains the usability of data for meta-analyses as they are often needed to calculate effect sizes and their associated sampling variance (e.g., log response ratio).

Here, we bridge the gaps in previous databases by systematically reviewing upper thermal limits of amphibian species. We focused on amphibians because 41% of the assessed species are threatened^39^, and temperature is an important driver of their extinction^40–42^. We compiled a comprehensive and representative sample of the literature through scoping various databases and assessing the literature in seven different languages to increase the representation of data from traditionally under-represented (and non-English speaking) countries^30^. We also included various forms of intraspecific variation (e.g., different acclimation temperatures, different life stages) to provide a more comprehensive and flexible picture of how amphibians cope with extreme temperatures. Finally, our data contain measures of dispersion for most (75%) estimates, which will facilitate the inclusion of these data in meta-analyses.

## Methods

### Literature searches

We aimed to obtain a comprehensive and representative sample of the published and unpublished experimental literature assessing the upper thermal limits of amphibian species. We accessed Scopus, ISI Web of Science (core collection), Lens, and ProQuest (dissertation & theses) on 2021/05/31 using The University of New South Wales’ institution subscriptions and did not apply a time span limit. We also obtained non-English language literature by conducting additional searches in Google Scholar in traditional Chinese, simplified Chinese, French, Japanese, Portuguese, and Spanish. We chose these languages to maximise the discovery of findings where amphibian species diversity is the highest^43^. We did not assess additional languages due to time constraints and excluded languages for which we found limited evidence during pilot searches. For instance, this included common Southeast Asian languages such as Malay, Bahasa Indonesia, and Vietnamese.

English search strings were adapted to the structure of each database (Supplementary Table S1), and we complemented our searches with backward searches, where we collected studies cited in previous compilations of ectotherm or amphibian upper thermal limits^2,15–17,24,25^. Our database and backward searches retrieved 1672 unique documents that were screened by PPottier for title, abstract and keyword content in Rayyan QCRI^44^. We used this software to highlight important keywords and filter publications based on our inclusion criteria. A total of 271 documents were then assessed for eligibility by PPottier (81.5%) and YY (18.5%) and 177 studies were found eligible.

**Fig. 1:**
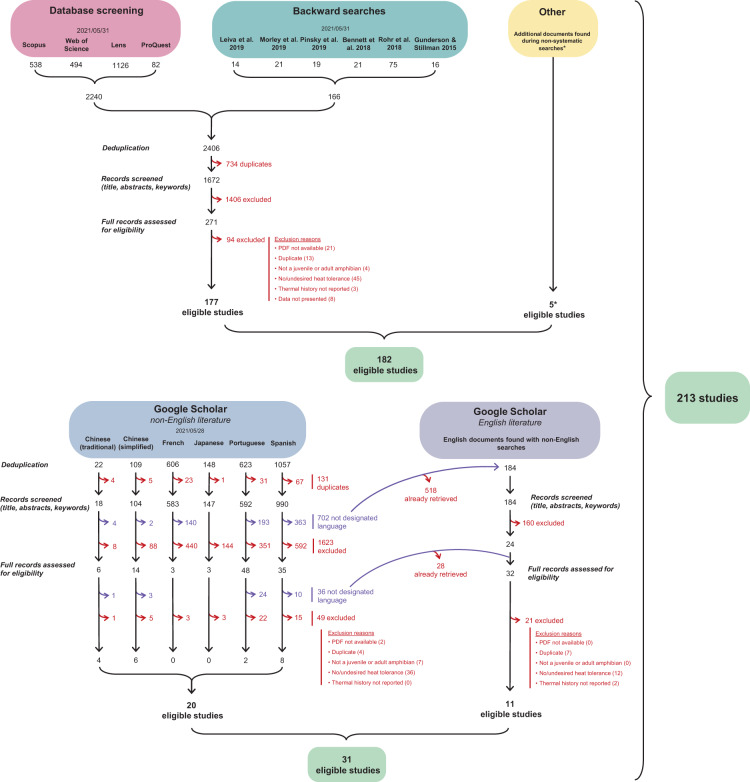
PRISMA flowchart. Highlighted are the different databases used, the number of studies included at each stage of the screening, and the reasons for excluding studies. *The five studies found during non-systematic searches include two studies found during pilot searches in Google, and three studies mistakenly excluded during the screening, but included in a previous synthesis by Rohr and colleagues^16^.

Translated searches were performed in Google Scholar because other databases provide poor indexing of studies published in languages other than English. Indeed, respectively 95.37% and 92.64% of the documents indexed in Scopus and Web of Science are in English^45^. Although Google Scholar suffers from reproducibility issues^46^, it is recommended as a complementary database to systematic reviews^47,48^. A suite of keywords was translated by native speakers: “*thermal tolerance*”, “*critical temperature*”, “*thermal limit*”, “*lethal temperature*”, “*amphibian*”, “*frog*”, “*toad*”, “*salamander*”, “*newt*”, “*tadpole*”. Each keyword (or combination of keywords) was translated in its singular and/or plural form, where applicable. Because Google Scholar’s search strings have a limitation of 256 characters, each keyword (or combination of keywords) was sequentially assessed in its singular or plural form, and the search returning the greatest hit rate was selected. All Google scholar searches had the form (“*thermal tolerance*” OR *synonyms*) AND (*amphibian* OR *synonyms*). For each language, we also performed a search where we replaced “*thermal tolerance”* by “*CTmax*”, a keyword used in various languages (Supplementary Table S1). Bibliographic records were extracted from Google Scholar using Publish or Perish^49^. We also found two additional studies in traditional Chinese^50,51^ during pilot searches in Google (benchmarking *sensu*^29^). NR (50%) and JOV (50%) performed the screening of articles in Spanish, while YY, RRYO, PPottier, SN, and PPollo screened documents in traditional Chinese, simplified Chinese, French, Japanese, and Portuguese, respectively. Twenty eligible studies in languages other than English were found following those searches. Moreover, we found 11 eligible studies published in English that were not retrieved by the English searches we performed (Fig. 1). Eventually, 14.6% of the included studies were retrieved through searches in languages other than English.

### Eligibility criteria

We focused on experimental studies that measured the upper thermal limit of wild and laboratory amphibians. We selected studies based on three main criteria. First, we only considered data on juveniles and adults. We excluded studies on embryonic traits because methods used to quantify their upper thermal limits tend to differ from those used for juveniles and adults. Second, we only included studies using the most reported measures of upper thermal limits, which are analogous to the fundamental thermal niche animals can exploit. Specifically, we included studies measuring the critical thermal maximum (CT_max_) or the median lethal temperature (LT_50_) of amphibians. CT_max_ represents a measure of upper thermal limit where the temperature is increased progressively until an endpoint is reached (e.g., loss of righting response^5^). Conversely, LT_50_ is assessed by subjecting animals to constant temperatures and measuring their survival after a given period^4^. The temperature lethal for 50% of the animals (LT_50_) is then interpolated from the survival data at different temperatures. We also considered studies reporting the time to death at different static temperatures because these data can be converted to CT_max_ estimates^52^. Because CT_max_ occurs before death and the conversion between static and dynamic CT_max_ relies on certain assumptions, the accuracy of our conversions needs statistical validation before inclusion in statistical analyses. We chose to focus on these proxies to maximise the comparability of estimates across species and therefore, the usability of the database. Third, because upper thermal limits are intrinsically plastic^14–17^, we only considered studies reporting the temperature at which animals were maintained in the laboratory (i.e., the temperature of acclimation), the temperature of the environment from which animals were captured (i.e., the temperature of acclimatization), or the geographical coordinates and dates of capture (which may allow the estimation of acclimatization temperatures). Decision trees and inclusion criteria are presented in Supplementary Figures S1-2 and Tables S3-4. Search methods and reasons for excluding studies are summarised in a PRISMA flowchart (Fig. 1). Ultimately, we retrieved 213 relevant publications^42,50,51,53–262^.

### Data extraction

We extracted the mean upper thermal limit of amphibians along with their associated sample sizes and measures of dispersion (i.e., standard deviation or standard error). Data extractions were performed by PPottier (50.0%), HYL (8.9%), RRYO (7.5%), ANRV (9.4%), PPollo (8.9%), JOV (8.9%) and YY (6.5%). Data from text and tables were directly extracted, while data presented in figures were digitised using the *metaDigitise* package^263^ (v. 1.0.1) in R^264^ (v. 4.1.3). We also calculated means, standard deviations, and sample sizes from the raw data, when available. When data were presented from different sources (e.g., table and figure), we favoured the source having the highest resolution (e.g., extract the data for each sex instead of the pooled data for both sexes). For studies only reporting the survival time at different temperatures, we converted these data to CT_max_ using the thermal tolerance landscape framework^6,52^. We performed a linear regression of the logarithm of the time to the death against the test temperatures, and interpolated the temperature amphibians could tolerate for 1 hour as a proxy for CT_max_ (following the recommendations of^52^). For the 85 studies included in the synthesis by Rohr and colleagues^16^, we extracted the data presented in this synthesis, and we completed missing cases using the original study. However, we re-extracted the data from tables, figures and/or raw data when data could be extracted at a finer resolution.

We also extracted other variables to account for potential sources of heterogeneity and non-independence in the data. Notably, we assigned unique identifiers for each estimate, study, species, population, and cohort to encourage quantifying the variance explained by those variables (see^34^). We included all sources of within-species variation presented in the original studies. This includes, for instance, data from experimental groups exposed to different acclimation temperatures, different life stages, different populations, or different endpoints used to infer upper thermal limits. The full list of extracted variables is described in Supplementary Table S2.

Species names and taxonomy were standardized according to the most comprehensive phylogenetic tree for amphibians to date^265^, which mostly aligns with AmphibiaWeb^266^. We also included data from species not described at the species level (e.g., *Noblella* sp.) and species not indexed in AmphibiaWeb^266^ or presented in^265^. We believe that this database will be updated, and therefore the taxonomic information may change, and be useful to future users. As our study focused on naturally occurring species, we did not include data from experimentally bred hybrid species.

Data were organised in a ‘stacked’ format (following the recommendations of^267^), to facilitate their use in comparative studies. Note that these data can be converted to an ‘effect size’ format for meta-analysis.

### Data curation

Because the data we extracted contains various sources of within-species variability, estimates may not be directly comparable for most comparative analyses. Therefore, we provided a curated version of the database, which excludes data having procedural concerns (see Supplementary Table S5); animals exposed to toxicants, hormones, or high levels of UV radiations; extinct species; and species not described at the species level. This subset of the data (197 studies, 587 species) is suitable for answering most questions in ecophysiology. A script detailing each step of the data curation is available via Zenodo^268^.

## Data Records

**Fig. 2:**
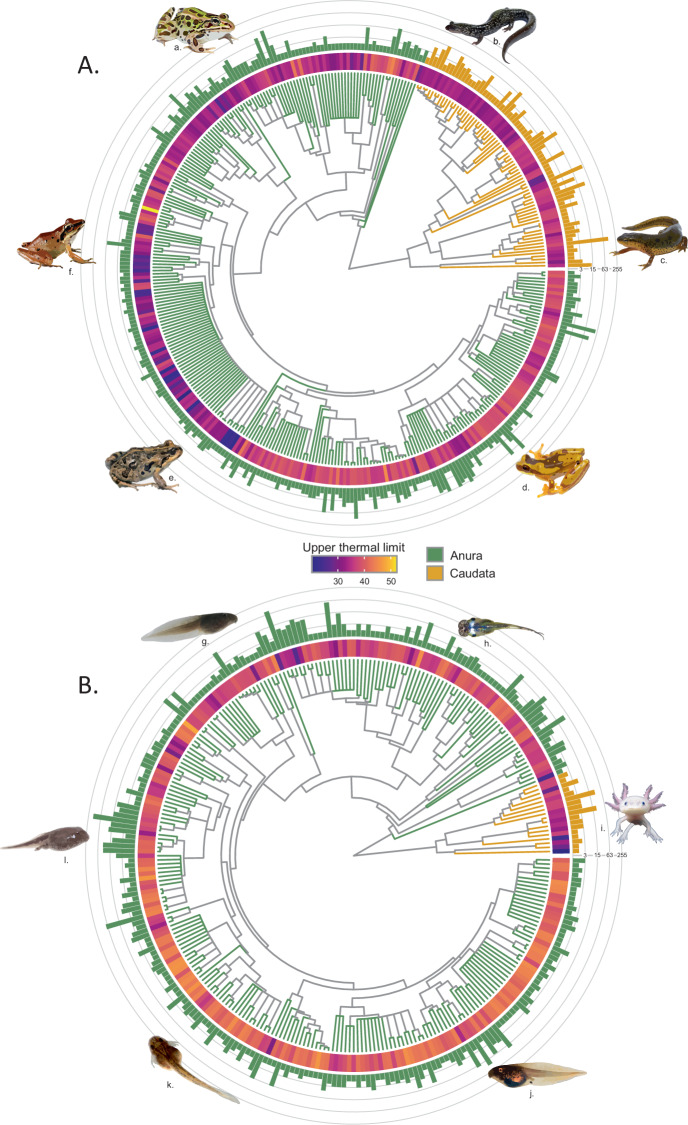
Distribution of estimates and mean upper thermal limits across the phylogeny of species included in the database. (**A**) adults. (**B**) larvae and juveniles. The number of estimates compiled per species (histograms) is presented on a log_2_(x + 1) scale. Phylogeny is based on the consensus of 10,000 fully-sampled trees from the posterior distribution, as described in^265^. a. *Rana pipiens* © Brian Gratwicke, b. *Plethodon cylindraceus* © Brian Gratwicke, c. *Notophthalmus viridescens* © Brian Gratwicke, d. *Dendropsophus ebraccatus* © Brian Gratwicke, e. *Pleurodema thaul* © Richard Sage, f. *Craugastor longirostris*, g. *Limnodynastes peronii* © Evan Pickett, h. *Microhyla heymonsi*, i. *Ambystoma mexicanum* © Tim Flach, j. *Hyla versicolor* © Michael F. Benard, k. *Engystomops pustulosus* © Daniel J. Paluh. l. *Anaxyrus americanus* © Brian Gratwicke.

**Fig. 3:**
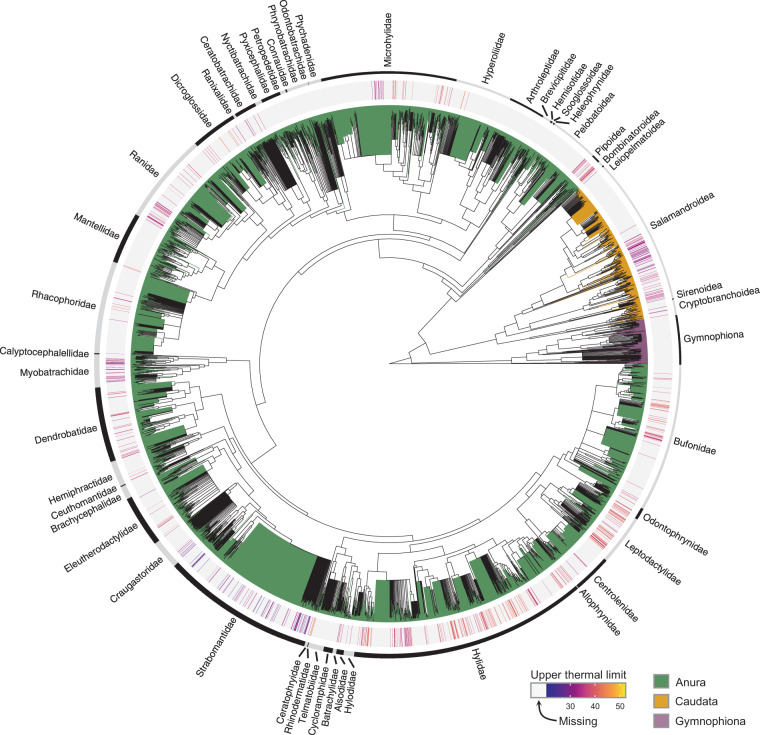
Distribution of estimates across the phylogeny of nearly all extant amphibians. The upper thermal limits of amphibian species included in our database are mapped on the phylogeny of nearly all extant species (7,238 species). Family names are presented in the outer circle, which was adapted from^265^. Phylogeny is based on the consensus of 10,000 fully-sampled trees from the posterior distribution, as described in^265^.

**Fig. 4:**
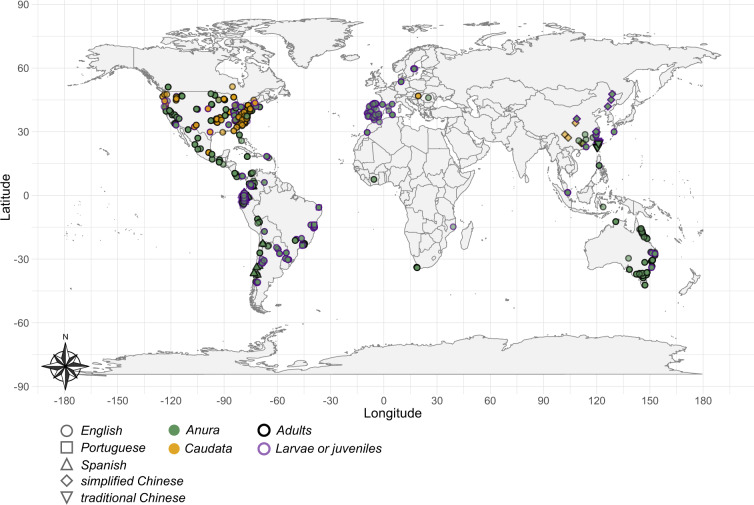
Geographical locations at which experimental data were collected. Points denote which order of amphibians were assayed (point filling), at which life stage (point border) and in what language were the findings published (point shape). Note that geographical coordinates were missing for 659 (21.3%) of the estimates, notably when animals were raised in the laboratory for numerous generations.

This database includes upper thermal limits of 616 amphibian species (~7% of all described species) across 37 families, with a broad geographical and phylogenetic coverage (Figs. 2–4). There are three key strengths of this database. First, it captures the substantial within-species variation, wherein 5.02 ± 12.6 (mean ± SD) upper thermal limit estimates have been extracted for each species on average (range: 1–163 estimates per species). Second, it formally includes data from non-English-language publications. In fact, 27 studies included in our synthesis were published in languages other than English, which represented 17 estimates taken from publications in Portuguese (9 species, 2 studies), 61 estimates from Spanish (37 species, 8 studies), 53 estimates from simplified Chinese (12 species, 10 studies) and 233 estimates from traditional Chinese publications (21 species, 6 studies). Third, our systematic review can easily be reproduced, updated, and expanded to a wider range of species, including other ectothermic taxa.

Our database is hosted in Github (https://github.com/p-pottier/Amphibian_upper_thermal_limits) and archived in Zenodo^268^. These repositories contain the (i) metadata (.csv), (ii) raw data (.csv), (iii) curated data (.csv), (iv) code detailing the data curation (.Rmd), (v) code for producing the figures in this data descriptor (.Rmd), (vi) supplementary data (.csv) and phylogenetic tree (.tre) used to build the figures in this data descriptor, and vii) a bibliographic file containing all the references cited in the database (.RIS). Data records are under a CC-BY license that enables reuse with attribution of this data descriptor, and we encourage the citation of original data sources.

## Technical Validation

We systematically reviewed >4000 studies in five databases, and seven languages, using a transparent and reproducible workflow. However, we acknowledge that searches in Google Scholar may not be entirely reproducible. In fact, Google Scholar’s updating practices are not publicly available^46^, and results may vary over time. However, Google Scholar is still deemed as a complementary source of evidence to systematic reviews^47,268^, and was the best available tool, to our knowledge, for accessing materials in languages other than English.

We are confident to have obtained a representative and nearly comprehensive sample of the literature on amphibian upper thermal limits. Comparisons with previously published databases show that our synthesis (213 studies retrieved from searches in five databases and seven languages) has greatly expanded the current state of knowledge in the field. For instance, the Globtherm database^24^ contains data for 113 species (20 studies retrieved from English searches in one database), whereas the synthesis by Rohr and colleagues^16^ contains data for 251 species (85 studies retrieved from undescribed searches). However, it is important to note that our database still suffers from both taxonomic and geographic biases in sampling (Figs. 3–4). Caecilians (Gymnophiona), an entire order of amphibians, are not represented in our data (Fig. 3). Furthermore, we found very little published evidence from high latitudes, across the African continent, and in most of Asia (Fig. 4). While amphibian species richness is expected to be lower at high latitudes, we expected to retrieve more data from species native to Africa and Asia, where amphibian diversity is high^43^. We encourage researchers possessing relevant data to contact us and/or post an issue in Github to update the database.

Different authors extracted the data from original studies, which may have introduced some errors. Therefore, all data were cross-checked and standardized by the data extraction leader, PPottier. Data were explored in R to identify mistakes that occurred during data extraction. Data that were deemed as having procedural concerns (e.g., unclear acclimation conditions, exposure to UV radiations) were flagged, and removed during data curation. We also extracted measures of data dispersion around mean estimates (i.e., standard deviation, standard error) to help infer which estimates are the most representative of population estimates. While 25.1% of the estimates had no measures of dispersion, these could be estimated using data imputation procedures e.g.^269,270^. We also extracted information about factors that may generate data heterogeneity, or potential confounders. We encourage researchers to evaluate the comparability of the estimates they use in comparative analyses (see Usage Notes).

## Usage Notes

We provide both the raw and curated versions of our database. For most questions in ecophysiology, we recommend using the curated database, as it does not contain animals exposed to hormones, chemicals, UV radiation, or pathogens; data having procedural concerns (e.g., unclear acclimation conditions, survival data converted to CT_max_ estimates); extinct species; and species not described at the species level. However, the raw data, or some subsets of it, could be used to address specific questions, such as whether chemical concentrations influence the capacity for amphibians to tolerate heat.

Our data also contains intrinsic sources of non-independence. We extracted multiple estimates per study, species, population, and cohort (e.g., multiple endpoints assessed on the same animals). Species also vary in terms of their evolutionary relatedness, and we recommend performing phylogenetically-informed syntheses. The compatibility of our database with the phylogeny from Jetz and Pyron^265^ should facilitate this task. We recommend using a hierarchical random effect structure to account for, quantify, and decompose the variance explained by those different components^34^.

Because upper thermal limits are plastic and vary with acclimation temperatures^14–18^, it is important to account for this variation in future studies. We recommend using this variable as a fixed factor and consider accounting for non-linear relationships between upper thermal limits and acclimation temperatures^10^. For a meta-analysis, one may convert the database to an ‘effect size’ format (*sensu* Schwanz *et al*.^267^) and use stepwise comparisons between acclimation temperatures as effect sizes^14,271^. Other variables affecting upper thermal limits include geographical origin^9,272^, ontogeny^11,14,273^, assay duration^274,275^, upper thermal limit metrics^10^, body size^16,26,35^, sex^271,276^, and photoperiod^111^. We recommend quantifying the variation arising from those variables and considering their inclusion in statistical models. Finally, we recommend weighing estimates by their precision to make the estimates more precise^34,37^, and more representative of the “true” populations sampled in statistical analysis (e.g., meta-analysis and comparative analysis). While not all our estimates contain a measure of dispersion, data imputation procedures can be used to retrieve missing standard deviations^269,270^.

Finally, we encourage users’ feedback to maintain the quality and usability of the database. We welcome the reporting of potential errors and encourage users possessing relevant data to update the database to increase its geographical and taxonomical coverage. User feedback can be issued at the GitHub repository (https://github.com/p-pottier/Amphibian_upper_thermal_limits).

## Supplementary information

Supplementary Information

## Data Availability

The code used to curate the data and produced the figures in this data descriptor are available at Zenodo^268^. All data manipulations were performed using the *R* statistical environment^6^ (v. 4.1.3) and the *tidyverse* package^277^ (v. 1.3.1). Figures were built upon the *ggspatial*^278^ (v. 1.1.5), *maps*^279^ (v. 3.3.0), *ggtree*^280^ (v. 3.1.1), *ggtreeExtra*^281^ (v. 1.3.3), *ggnewscale*^282^ (v. 0.4.5), *patchwork*^283^ (v. 1.1.1), and *ape*^284^ (v. 5.5) packages.
