# Supplementary material for: A comprehensive database of amphibian heat tolerance
Source: Sci Data. 2022 Oct 4;9:600. doi: 10.1038/s41597-022-01704-9 (PMC9532409; doi:10.1038/s41597-022-01704-9)
Supplement: Supplementary file 1 — Supplementary Information [file 41597_2022_1704_MOESM1_ESM.pdf]

Supplementary Information

TABLE OF CONTENTS

Figure S1 2
Figure S2 3
Table S1 4
Table S2 8
Table S3 14
Table S4 15
Table S5 16

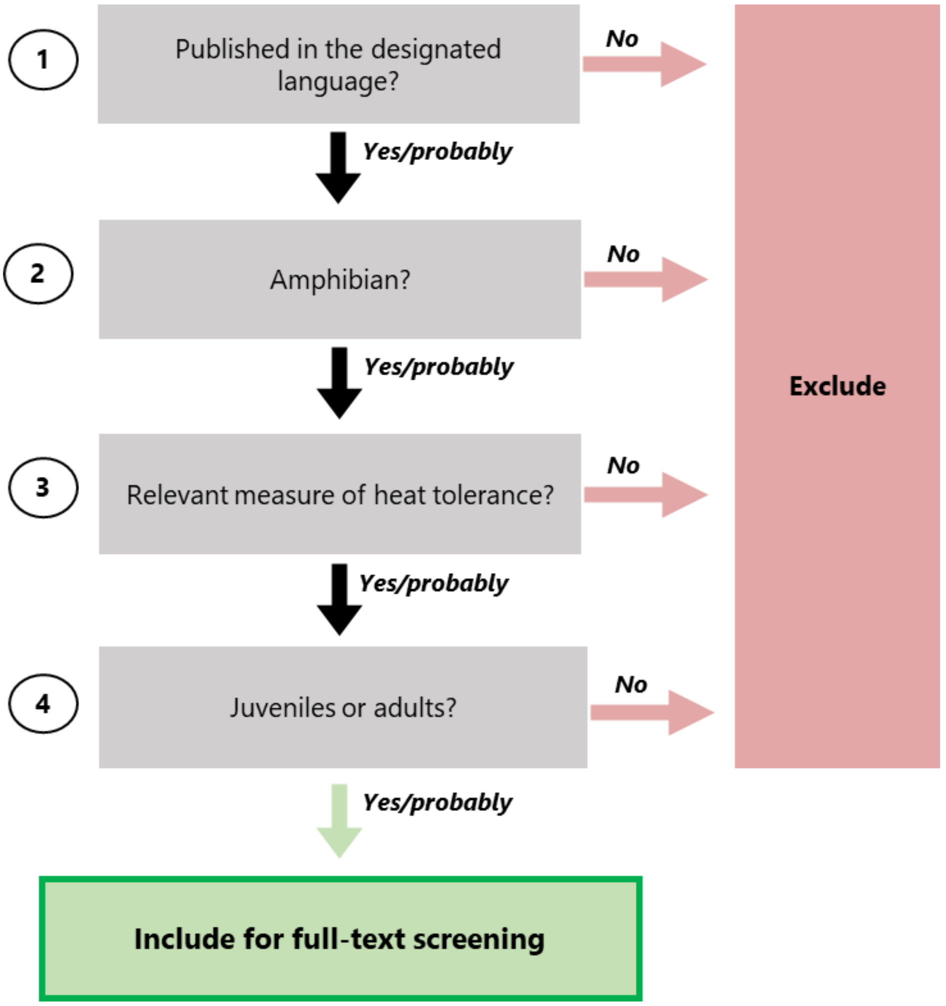

Figure S1: Decision tree used to screen titles, abstracts, and keywords. Additional details can be found in Table S3.

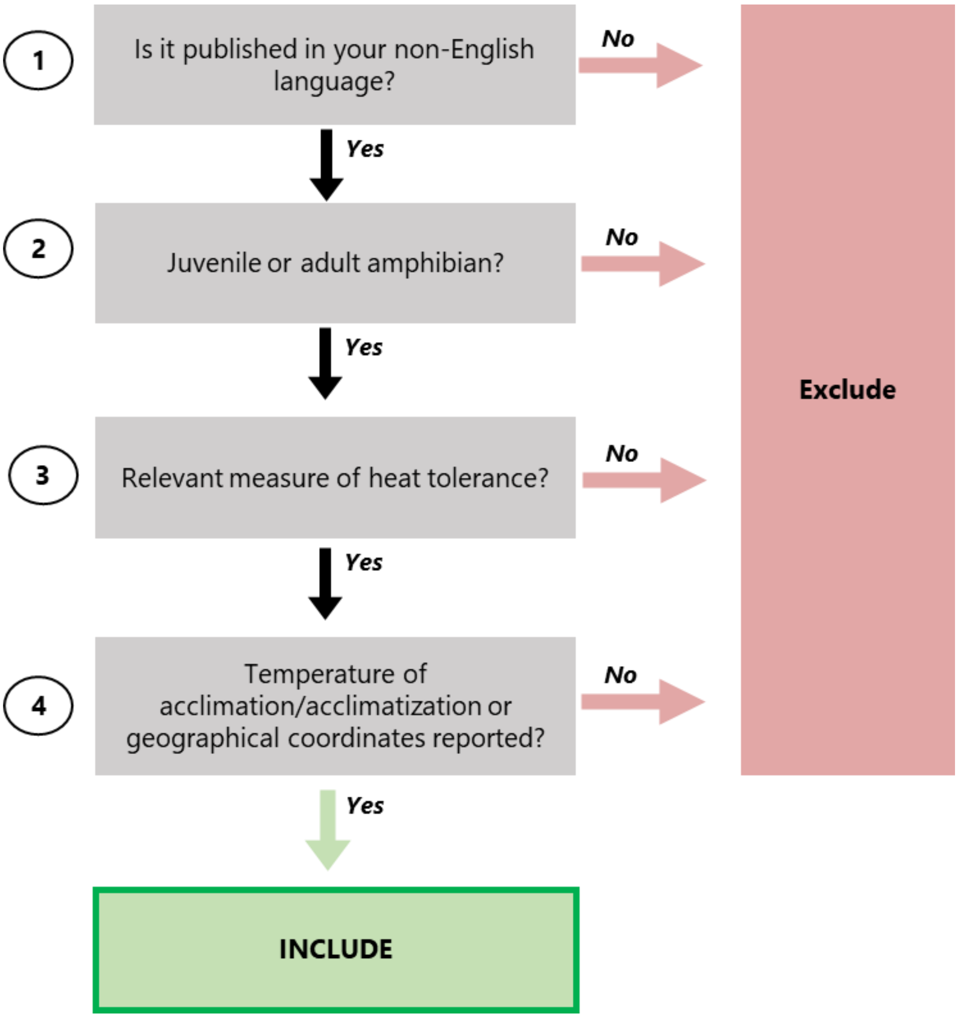

Figure S2: Decision tree used to assess full articles for eligibility. Additional details can be found in Table S4.

Table S1: Search strings used for the different databases.

**Database**	**Search strings**
Scopus	TITLE-ABS-KEY("temperature*" OR "thermal" OR “cold*” OR “cool*” OR “heat*” OR “hot” OR “warm*”) AND TITLE-ABS-KEY("thermal tolerance*" OR "thermotolerance*" OR "heat tolerance*" OR "tolerance* to heat*" OR "tolerance* to temperature*" OR "temperature* tolerance*" OR "physiological tolerance*" OR "thermal m*" OR "critical temperature*" OR "thermal limit*" OR "thermal breadth*" OR "tolerance breadth*" OR "thermal range*" OR "thermal window*" OR "tolerance window*" OR "warming tolerance*" OR "tolerance* to warming" OR "CTmax" OR "CT max" OR "heat coma" OR "lethal temperature*" OR "temperature* lethal" OR "lethal limit*" OR "LTmax" OR "LT max" OR "heat stress tolerance*" OR "tolerance to heat stress" OR "thermal stress tolerance*" OR "tolerance* to thermal stress" OR "temperature stress tolerance*" OR "tolerance* to temperature stress" OR "tolerance* to heat shock " OR "heat shock tolerance*" OR "loss of equilibrium" OR "onset of spasm*" OR "loss of righting response" OR "heat stupor" OR "death point*" OR "LT50" OR "LT 50" OR "LT100" OR "LT 100" OR "ULT50" OR "ULT 50" OR "ULT100" OR "ULT 100" OR "UILT" OR "UILT50" OR "UILT 50" OR "heat knockdown" OR "heat knock-down" OR "knockdown resistance" OR "knock-down resistance" OR "death time*" OR "knockdown time*" OR "knock-down time" OR "lethal time*" OR "heat resistance" OR "resistance to heat stress" OR "knockdown temperature*" OR "knock-down temperature*") AND TITLE-ABS-KEY("amphibia*" OR "*frog*" OR "toad*" OR "salamand*" OR "newt" OR "newts" OR "tadpole*" OR "metamorph" OR "metamorphs" OR "caecili*" OR "rhinatrema*" OR "ichthyophi*" OR "scolecomorph*" OR "chikil*" OR "herpelidae" OR "typhlonect" OR "indotyphlid*" OR "dermophi*" OR "siphonop*" OR "caudata" OR "urodela" OR "cryptobranch*" OR "hynobiid*" OR "sirenidae" OR "ambystoma*" OR "dicamptodon*" OR "proteidae" OR "rhyacotriton*" OR "amphium*" OR "plethodon*" OR "anura*" OR "ascaph*" OR "leiopelma*" OR "bombina*" OR "alyt*" OR "rhinophryn*" OR "pipidae" OR "xenopus" OR "scaphiop*" OR "pelodyt*" OR "megophry*" OR "pelobat*" OR "heleophryn*" OR "calyptocephalell*" OR "myobatrach*" OR "rhinoderma*" OR "alsod*" OR "hylod*" OR "batrachyl*" OR "cycloramph*" OR "telmatob*" OR "ceratophry*" OR "hemiphract*" OR "hyla*" OR "hylidae" OR "bufo*" OR "leptodactyl*" OR "odontophryn*" OR "allophryn*" OR "centrolen*" OR "dendrobat*" OR "ceuthomanti*" OR "eleutherodactyl*" OR "brachycephalidae" OR "craugastor*" OR "strabomantidae" OR "pristimantis" OR "nasikabatrach*" OR "soogloss*" OR "microhyl*" OR "arthroleptid*" OR "hyperol*" OR "brevicipitidae" OR "hemisus" OR "odontobatrach*" OR "phrynobatrach*" OR "ptychaden*" OR "conraua" OR "petropedet*" OR "pyxicephal*" OR "micrixalus" OR "nyctibatrach*" OR "ranixalidae" OR "ceratobatrach*" OR "dicroglossidae" OR "rana" OR "ranidae" OR "rhacophor*" OR "mantellidae")
Web of Science (core collection)	TS=("temperature*" OR "thermal" OR “cold*” OR “cool*” OR “heat*” OR “hot” OR “warm*”) AND TS=("thermal tolerance*" OR "thermotolerance*" OR "heat tolerance*" OR "tolerance* to heat*" OR "tolerance* to temperature*" OR "temperature* tolerance*" OR "physiological tolerance*" OR "thermal m*" OR "critical temperature*" OR "thermal limit*" OR "thermal breadth*" OR "tolerance breadth*" OR "thermal range*" OR "thermal window*" OR "tolerance window*" OR "warming tolerance*" OR "tolerance* to warming" OR "CTmax" OR "CT max" OR "heat coma" OR "lethal temperature*" OR "temperature* lethal" OR "lethal limit*" OR "LTmax" OR "LT max" OR "heat stress tolerance*" OR "tolerance to heat stress" OR "thermal stress tolerance*" OR "tolerance* to thermal stress" OR "temperature stress tolerance*" OR "tolerance* to temperature stress" OR "tolerance* to heat shock " OR "heat shock tolerance*" OR "loss of equilibrium" OR "onset of spasm*" OR "loss of righting response" OR "heat

	stupor" OR "death point*" OR "LT50" OR "LT 50" OR "LT100" OR "LT 100" OR "ULT50" OR "ULT 50" OR "ULT100" OR "ULT 100" OR "UILT" OR "UILT50" OR "UILT 50" OR "heat knockdown" OR "heat knock-down" OR "knockdown resistance" OR "knock-down resistance" OR "death time*" OR "knockdown time*" OR "knock-down time" OR "lethal time*" OR "heat resistance" OR "resistance to heat stress" OR "knockdown temperature*" OR "knock-down temperature*") AND TS=("amphibia*" OR "*frog*" OR "toad*" OR "salamand*" OR "newt" OR "newts" OR "tadpole*" OR "metamorph" OR "metamorphs" OR "caecili*" OR "rhinatrema*" OR "ichthyophi*" OR "scolecomorph*" OR "chikil*" OR "herpelidae" OR "typhlonect" OR "indotyphlid*" OR "dermophi*" OR "siphonop*" OR "caudata" OR "urodela" OR "cryptobranch*" OR "hynobiid*" OR "sirenidae" OR "ambystoma*" OR "dicamptodon*" OR "proteidae" OR "rhyacotriton*" OR "amphium*" OR "plethodon*" OR "anura*" OR "ascaph*" OR "leiopelma*" OR "bombina*" OR "alyt*" OR "rhinophryn*" OR "pipidae" OR "xenopus" OR "scaphiop*" OR "pelodyt*" OR "megophry*" OR "pelobat*" OR "heleophryn*" OR "calyptocephalell*" OR "myobatrach*" OR "rhinoderma*" OR "alsod*" OR "hylod*" OR "batrachyl*" OR "cycloramph*" OR "telmatob*" OR "ceratophry*" OR "hemiphract*" OR "hyla*" OR "hylidae" OR "bufo*" OR "leptodactyl*" OR "odontophryn*" OR "allophryn*" OR "centrolen*" OR "dendrobat*" OR "ceuthomanti*" OR "eleutherodactyl*" OR "brachycephalidae" OR "craugastor*" OR "strabomantidae" OR "pristimantis" OR "nasikabatrach*" OR "soogloss*" OR "microhyl*" OR "arthroleptid*" OR "hyperol*" OR "brevicipitidae" OR "hemisus" OR "odontobatrach*" OR "phrynobatrach*" OR "ptychaden*" OR "conraua" OR "petropedet*" OR "pyxicephal*" OR "micrixalus" OR "nyctibatrach*" OR "ranixalidae" OR "ceratobatrach*" OR "dicroglossidae" OR "rana" OR "ranidae" OR "rhacophor*" OR "mantellidae")
Lens	("temperature*" OR "thermal" OR “cold*” OR “cool*” OR “heat*” OR “hot” OR “warm*”) AND ("thermal tolerance*" OR "thermotolerance*" OR "heat tolerance*" OR "tolerance* to heat*" OR "tolerance* to temperature*" OR "temperature* tolerance*" OR "physiological tolerance*" OR "thermal m*" OR "critical temperature*" OR "thermal limit*" OR "thermal breadth*" OR "tolerance breadth*" OR "thermal range*" OR "thermal window*" OR "tolerance window*" OR "warming tolerance*" OR "tolerance* to warming" OR "CTmax" OR "CT max" OR "heat coma" OR "lethal temperature*" OR "temperature* lethal" OR "lethal limit*" OR "LTmax" OR "LT max" OR "heat stress tolerance*" OR "tolerance to heat stress" OR "thermal stress tolerance*" OR "tolerance* to thermal stress" OR "temperature stress tolerance*" OR "tolerance* to temperature stress" OR "tolerance* to heat shock " OR "heat shock tolerance*" OR "loss of equilibrium" OR "onset of spasm*" OR "loss of righting response" OR "heat stupor" OR "death point*" OR "LT50" OR "LT 50" OR "LT100" OR "LT 100" OR "ULT50" OR "ULT 50" OR "ULT100" OR "ULT 100" OR "UILT" OR "UILT50" OR "UILT 50" OR "heat knockdown" OR "heat knock-down" OR "knockdown resistance" OR "knock-down resistance" OR "death time*" OR "knockdown time*" OR "knock-down time" OR "lethal time*" OR "heat resistance" OR "resistance to heat stress" OR "knockdown temperature*" OR "knock-down temperature*") AND ("amphibia*" OR "*frog*" OR "toad*" OR "salamand*" OR "newt" OR "newts" OR "tadpole*" OR "metamorph" OR "metamorphs" OR "caecili*" OR "rhinatrema*" OR "ichthyophi*" OR "scolecomorph*" OR "chikil*" OR "herpelidae" OR "typhlonect" OR "indotyphlid*" OR "dermophi*" OR "siphonop*" OR "caudata" OR "urodela" OR "cryptobranch*" OR "hynobiid*" OR "sirenidae" OR "ambystoma*" OR "dicamptodon*" OR "proteidae" OR "rhyacotriton*" OR "amphium*" OR "plethodon*" OR "anura*" OR "ascaph*" OR "leiopelma*" OR "bombina*" OR "alyt*" OR "rhinophryn*" OR "pipidae" OR "xenopus" OR "scaphiop*" OR "pelodyt*" OR "megophry*" OR "pelobat*" OR "heleophryn*" OR "calyptocephalell*" OR "myobatrach*" OR

	"rhinoderma*" OR "alsod*" OR "hylod*" OR "batrachyl*" OR "cycloramph*" OR "telmatob*" OR "ceratophry*" OR "hemiphract*" OR "hyla*" OR "hylidae" OR "bufo*" OR "leptodactyl*" OR "odontophryn*" OR "allophryn*" OR "centrolen*" OR "dendrobat*" OR "ceuthomanti*" OR "eleutherodactyl*" OR "brachycephalidae" OR "craugastor*" OR "strabomantidae" OR "pristimantis" OR "nasikabatrach*" OR "soogloss*" OR "microhyl*" OR "arthroleptid*" OR "hyperol*" OR "brevicipitidae" OR "hemisus" OR "odontobatrach*" OR "phrynobatrach*" OR "ptychaden*" OR "conraua" OR "petropedet*" OR "pyxicephal*" OR "micrixalus" OR "nyctibatrach*" OR "ranixalidae" OR "ceratobatrach*" OR "dicroglossidae" OR "rana" OR "ranidae" OR "rhacophor*" OR "mantellidae")<br><br>With filters: Field of Study = (excl Physics , excl Particle physics , excl Quantum mechanics , excl Condensed matter physics , excl Phase transition , excl Quantum electrodynamics , excl Quantum chromodynamics , excl Materials science , excl Mathematical physics , excl Superconductivity , excl Theoretical physics , excl Electroweak interaction , excl Fermion , excl Cell biology , excl Quark , excl Higgs boson , excl Thermal quantum field theory , excl Botany , excl Geology , excl Lattice (order) , excl Lattice field theory , excl Statistical physics , excl Gauge theory , excl MAJORANA , excl Baryogenesis , excl Biophysics , excl Scalar (mathematics) , excl Order (ring theory) , excl Symmetry breaking , excl Agronomy , excl Quantum , excl Ising model , excl Phase (matter) , excl Field (physics) , excl Lattice QCD , excl Coupling constant , excl Effective field theory , excl Meson , excl Quark–gluon plasma , excl Context (language use) , excl Magnetic field , excl Spontaneous symmetry breaking , excl Boson , excl Pion , excl Sigma model , excl Phase diagram , excl Quantum field theory , excl Scaling , excl Superfluidity , excl Lattice gauge theory , excl Astrophysics , excl Propagator , excl Topology , excl Baryon asymmetry , excl Dark matter , excl Quasiparticle , excl Critical exponent , excl Chiral perturbation theory , excl Standard Model , excl Fishery , excl Renormalization group , excl Renormalization , excl Critical phenomena , excl Pairing)
Proquest (Dissertation and Theses)	(noft(thermal tolerance*) OR noft(temperature* tolerance*) OR noft(thermal limit*) OR noft(heat tolerance*)) AND (noft(amphibia*) OR noft(frog*) OR noft(toad*) OR noft(anura*) OR noft(tadpole*) OR noft(salamand*) OR noft(newts))
Google Scholar (French)	("tolérance thermique" OR “température critique” OR “limite thermique” OR “température létale”) AND (amphibiens OR grenouille OR crapaud OR salamandres OR triton OR têtards OR Amphibia OR Caudata OR Anura OR batracien OR anoure)<br><br>CTmax AND (“amphibiens” OR grenouille OR crapaud OR “salamandres” OR triton OR têtards OR batracien OR anoure)
Google Scholar (Japanese)	(耐熱性 OR 臨界温度 OR 高温限界 OR 致死温度) AND (両生類 OR カエル OR ヒキガエル OR サンショウウオ OR イモリ OR オタマジャクシ OR Amphibia OR Caudata OR Anura)<br><br>CTmax AND (両生類 OR カエル OR ヒキガエル OR サンショウウオ OR イモリ OR オタマジャクシ)

Google Scholar (Portuguese)	(“tolerância térmica” OR “temperatura crítica” OR “limites térmicos” OR “temperatura letal”) AND (anfíbio OR “rã” OR sapos OR salamandra OR tritão OR girino OR Amphibia OR Caudata OR Anura OR anuros)<br><br>CTmax AND (anfíbio OR “rã” OR sapos OR salamandra OR tritão OR girino)
Google Scholar (simplified Chinese)	(耐热性 OR 临界温度 OR 热上线 OR 致死温度) AND (两栖动物 OR 青蛙 OR 蛤蟆 OR 蝾螈 OR 蝌蚪 OR 小鲵 OR 大鲵 OR Amphibia OR Caudata OR Anura)<br><br>(CTmax) AND (两栖动物 OR 青蛙 OR 蛤蟆 OR 蝾螈 OR 蝌蚪 OR 小鲵 OR 大鲵)
Google Scholar (traditional Chinese)	(溫度耐受 OR 臨界溫度 OR 溫度限制 OR 致死溫度) AND (兩棲類 OR 蛙青蛙 OR 蟾蟾蜍癩蝦蟆 OR 螈蠑螈OR 蝌蚪 OR 鯢小鯢山椒魚 OR 鯢大鯢娃娃魚 OR Amphibia OR Caudata OR Anura)<br><br>CTmax AND (兩棲類 OR 蛙青蛙 OR 蟾蟾蜍癩蝦蟆 OR 螈蠑螈 OR 蝌蚪 OR 鯢小鯢山椒魚 OR 鯢大鯢娃娃魚)
Google Scholar (Spanish)	(“tolerancia térmica” OR “temperatura crítica” OR “límites térmicos” OR “temperaturas letales”) AND (anfibio OR rana OR sapo OR salamandra OR tritón OR renacuajo OR Amphibia OR Caudata OR Anura OR anuros)<br><br>CTmax AND (anfibio OR rana OR sapo OR salamandra OR tritón OR renacuajo or anuros)

Table S2: Metadata.

Data	Description
name	Name of the researcher who performed the data extraction.
ref	Abbreviated reference for the study.
title	Title of the paper or thesis.
pub_year	Publication year of the paper or thesis.
thesis_chapter	If the study is a thesis, the chapter the data is taken from (e.g., 2). Note that when more than one chapter was relevant, a different study_ID (see below) was assigned to each chapter.
chapter_title	The title of the thesis chapter the data is taken from.
peer-reviewed	Whether the study was peer-reviewed or not (i.e., thesis). Factor with two levels: “peer-reviewed”, “not_peer-reviewed”.
doi	DOI of the paper.
language	Language of the paper (main text). Factor with seven levels: “English”, “traditional Chinese”, “simplified Chinese”, “French”, “Japanese”, “Portuguese”, “Spanish”.
screening_cat	Whether the study was found in the main database searches (Scopus, Web of Science, Lens, Proquest, backward searches), or in Google Scholar. Factor with two levels: “database”, “GS”.
row_n	Row number to order data.
es_ID	Unique identifier for each upper thermal limit estimate (e.g., es1, es2, es3).
study_ID	Unique identifier for each study (e.g., st1, st2, st3).
species_ID	Unique identifier for each species (e.g., sp1, sp2, sp3).
population_ID	Unique identifier for each population (e.g., pop1, pop2, pop3). We refer to distinctions between populations made by the authors (e.g., “northern population”, “central population”, and “southern population”).
cohort_ID	Unique identifier for each cohort (e.g., co1, co2, co3). By “cohort”, we refer to independent groups of animals. In some cases, upper thermal limits can be measured multiple times on the same cohort of animals (e.g., using different endpoints, or at different ages). As such, the same cohort_ID was assigned to repeated measures (e.g., co4, co4).
notes_ID	General notes related to es_ID, study_ID, species_ID, and population_ID.
order	Species order, according to AmphibiaWeb[266].

family	Species family, according to AmphibiaWeb[266].
species	Species name, according to AmphibiaWeb[266]. .
strain	The strain, variety, subspecies, or morph of the species, as reported in the study.
IUCN_status	International Union for the Conservation of Nature (IUCN) status, according to AmphibiaWeb[266].
notes_species	General notes related to the species taxonomy and IUCN status.
origin	Origin of studied animals. Factor with four levels: recently collected from the wild (i.e., "wild"), eggs laid in the laboratory (i.e., "lab"), animals provided from a supplier (i.e., "supplier") or "unclear". For studies collecting eggs from the wild and testing the same generation of animals, animals were considered as "wild".
n_generations_lab	Number of generations spent in the laboratory, if reported in the study.
latitude	Latitude from which animals were collected (decimal degrees). Latitudes presented in degrees/minutes/seconds were converted to decimal degrees. When geographical coordinates were not presented, the coordinates were estimated using Google Maps.
longitude	Longitude from which animals were collected (decimal degrees). Longitudes presented in degrees/minutes/seconds were converted to decimal degrees. When geographical coordinates were not presented, the coordinates were estimated using Google Maps.
elevation	Elevation from which animals were collected (meters above sea level), as reported in the study. When not reported, elevation was estimated using latitude and longitude and freemaptools.com.
date_sampling	Date at which the animals were sampled (format YEAR/MONTH/DAY, e.g., "2020/07/26").
month_sampling	Month from which the animals were collected.
year_sampling	Year from which the animals were collected.
start_range_sampling_dates	The beginning of the range of dates over which animals were collected. Indicated are both the month and the year of collection (e.g., "January_2015").
end_range_sampling_dates	The end of the range of dates over which animals were collected. Indicated are both the month and the year of collection (e.g., "September_2015").

comment_sampling	Additional comments regarding the sampling of the animals.
ambient_temp	For animals recently sampled from the wild (eggs not laid in the laboratory), the mean ambient temperature (°C) in the month of collection, if reported in the study. If animals were collected over a range of months, the mean temperature across this sampling period was reported.
substrate_temp	For animals recently sampled from the wild (eggs not laid in the laboratory), the mean temperature of the substrate (°C) in the month of capture. If animals were collected over a range of months, the mean temperature across this sampling period was reported.
water_temp	For animals recently sampled from the wild (eggs not laid in the laboratory), the mean water temperature (°C) in the month of collection. If animals were collected over a range of months, the mean temperature across this sampling period was reported.
field_body_temp	For animals recently sampled from the wild (eggs not laid in the laboratory), the mean body temperature (°C) measured in the field when animals were collected. If animals were collected over a range of months, the mean temperature across this sampling period was reported.
notes_env_temp	General notes regarding the sampling of animals in the field.
acclimated	Whether the animals were maintained in the laboratory for >12h or tested shortly after collection. Factor with two levels: "acclimated" or "field-fresh".
incubation_temp	For animals born in the laboratory, the mean temperature (°C) at which the embryos were incubated.
sd_incubation_temp	Variability (standard deviation) in incubation_temp (°C).
life_stage_acclimated	For acclimated animals, the life stage acclimated prior to the upper thermal limit assessment. Factor with three levels: "embryos_and_larvae", "juveniles" or "adults". Larval stages of salamanders and tadpoles are referred to as "larvae". Froglets and toadlets (post-metamorphic frogs or toads that did not reach sexual maturity) are referred to as "juveniles".
gosner_acclimated	For acclimated animals, the Gosner stage when the acclimation started, if reported in the study.
acclimation_temp	For acclimated animals, the mean temperature of acclimation (°C). Note that "acclimation" refers to a prolonged (>12h) exposure to a new temperature. Therefore, cold/heat shocks or housing conditions just prior to assessing upper thermal limits (e.g., 2 hours at 25°C) are not considered as "acclimation"

	conditions. If animals were exposed to multiple acclimation conditions (e.g., 15°C for 1 month, and then re-acclimated to 25°C for 7 days), we took the latest acclimation condition as the "acclimation_temp".
sd_acclimation_temp	Variability (standard deviation) in acclimation_temp (°C).
acclimation_time	The duration of acclimation (days).
notes_acclimation	General notes regarding the laboratory acclimation of animals.
life_stage_tested	The life stage tested for upper thermal limits. Factor with three levels: "larvae", "juveniles" or "adults". Larval stages of salamanders and tadpoles are referred to as "larvae". Froglets and toadlets (post-metamorphic frogs or toads that did not reach sexual maturity) are referred to as "juveniles".
gosner_tested	Gosner stage when the animals were assessed for upper thermal limits.
SVL	Mean snout-vent length of the animals (mm) when assessed for upper thermal limits, if reported in the study. Note that SVL data was often taken from Rohr et al. (2018).
body_mass	Mean body mass of the animals (g) when assessed for upper thermal limits.
age_tested	The age (days-post-hatching) at which the animals were tested for upper thermal limits.
sex	The sex of the animals. Factor with four levels: "male", "female", "mixed", "unknown". The "mixed" category was used when authors clearly stipulate that they mixed males and females.
metric	The metric used to assess thermal tolerance (CTmax, LT50 or heat-knockdown time). Factor with two levels: "CTmax", "LT50".
endpoint	The endpoint that was used for assessing upper thermal limits (loss of righting response, loss of equilibrium, onset of spasms, death, other). Factor with five levels: "LRR", "LOE", "OS", "death", "other". If "other", details are reported in "notes_test" (see below).
medium_test_temp	Whether the temperature measured during the test was the ambient, the water, or the body temperature. Factor with three levels: "ambient", "water", "body".
start_temp	If the metric was CTmax, the starting temperature used in the upper thermal limit assay (°C).
ramping	If the metric was CTmax, the ramping (heating) rate applied to the animals (°C/min).

set_time	If the metric was LT50, the time the animals spent at the test temperature (the time after which the animals the survival was assessed, in hours). If the authors report e.g., 96h-LT50, then set_time would be 96.
n_test_temp	If the metric was LT50, the number of temperatures tested to assess upper thermal limits. E.g., if authors measured survival at 36, 38, 39, and 41°C, n_test_temp = 4.
n_replicates_per_temp	If the metric was LT50, the number of replicates used at each test temperatures. E.g., if authors used 5 test temperatures and measured the survival of three independent cohorts of animals at each test temperature, then n_replicates_per_temp = 3.
n_animals_per_replicate	If the metric was LT50, the number of animals in each replicate.
n_test	General notes regarding the CTmax or LT50 assays.
humidity	Humidity at which animals were acclimated or tested (% relative humidity). If the humidity during the acclimation and the test were different, priority was given to the conditions of the test.
oxygen	Oxygen at which animals were acclimated or tested ($mg.L^{-1}$ dissolved oxygen). If the oxygen concentration during the acclimation and the test were different, priority was given to the conditions of the test.
salinity	Salinity at which animals were acclimated or tested (parts per thousands). If the salinity during the acclimation and the test were different, priority was given to the conditions of the test.
pH	pH at which animals were acclimated. If the pH during the acclimation and the test were different, priority was given to the conditions of the test.
photoperiod	Photoperiod at which animals were acclimated (number of hours of light per day).
chemical	If any, which chemical (e.g., pollutant, toxin) was added to the animals' environment. If animals were in a control group (i.e., only supplemented with a solvent), "control" was indicated.
hormone	If any, which hormone (e.g., corticosterone, thyroid hormone) was added to the animals' environment. If animals were in a control group (i.e., only supplemented with a solvent), "control" was indicated.
concentration_chemical_hormone	If any, the concentration of the hormones or chemicals used. If animals were in a control group, "0" was indicated.
unit_chemical_hormone	The unit used to quantify the chemical or hormonal concentration administered (e.g., g/L, ng/g of sediment).

infected	Whether the animals were infected with a pathogen. Indicate “infected” if the animals were infected with a pathogen. Otherwise, leave the field blank.
pathogen	If the animals were infected with a pathogen, the name of the pathogen (e.g., *Batrachochytrium dendrobatidis*).
notes_supplements	General notes regarding the addition of chemicals, hormones, or pathogens.
data_source	Where the upper thermal limit data is reported (main text, table, figure, published data).
data_url	If the data was published in a repository, the url link to the repository containing the data.
flag	Whether the study has procedural concerns (with details).
mean_UTL	Mean upper thermal limit of the animals tested (°C).
error_UTL	Standard deviation or standard error of mean_UTL (see error_type)
n_UTL	Sample size of mean_UTL. When the metric was LT50, the sample size was taken as the number of test temperatures (“n_test_temp”) * the number of replicates per test temperature (n_replicates_per_temp).
error_type	Whether the error is presented as standard deviations (i.e., “sd”) or standard errors (i.e., “se”).
notes_UTL	General notes about upper thermal limit estimates.

TABLE S3*: Inclusion criteria used to screen abstracts, titles and keywords.* Numbers match those used in in Figure S1 (decision tree).

	**Description**
1	Studies not published in French, Japanese, Portuguese, simplified Chinese, traditional Chinese or Spanish were excluded.
2	"Amphibians" refer to frogs, toads, salamanders, newts, and caecilians. We only included studies on whole organisms.
3	Desired measures of heat tolerance include the:<br>i) Critical thermal maximum (CTmax), where animals are subject to incremental increases in temperature until an endpoint (e.g., loss of equilibrium) is reached;<br>ii) The temperature lethal for 50% or 100% of the animals (LT50 or LT100; sometimes referred to as the "incipient lethal temperature" or "median lethal temperature), where survival is recorded after animals are abruptly transferred to a set of high temperatures for a given period of time (e.g., 24 hours) and LT50 is interpolated from the survival curve; and<br>iii) The death time or (or heat knockdown time) where animals are abruptly transferred to elevated temperatures and the time needed for animals to reach an endpoint (e.g., immobilisation, death) is recorded as the response. With the latter measure, the thermal tolerance limit can be inferred from the relationship between the time to death and the temperature of the knockdown assay. Therefore, death times must have been measured at >2 temperatures (e.g., lethal times at 38, 40, and 42°C).<br>We exclude alternative measures of heat tolerance which cannot be converted to the temperature scale (e.g., heat coma recovery time) or CTmax extrapolated from physiological performance curves (e.g., critical temperature for ATPase activity).
4	We focused our search on juveniles (i.e., tadpole, metamorph, froglet) or adults. Hence, we exclude studies only measuring the heat tolerance of embryos.

TABLE S4*: Inclusion criteria used to assess full articles for eligibility.* Numbers match those used in in Figure S2 (decision tree).

	Description
1	Studies not published in French, Japanese, Portuguese, simplified Chinese, traditional Chinese or Spanish were excluded.
2	“Amphibians” refer to frogs, toads, salamanders, newts, and caecilians. We only included studies on juveniles (i.e., tadpole, metamorph, froglet) or adults. Hence, we excluded studies only measuring the heat tolerance of embryos.
3	Desired measures of heat tolerance include the:<br>i) Critical thermal maximum (CTmax), where animals are subject to incremental increases in temperature until an endpoint (e.g., loss of equilibrium) is reached;<br>ii) The temperature lethal for 50% or 100% of the animals (LT50 or LT100; sometimes referred to as the “incipient lethal temperature” or “median lethal temperature), where survival is recorded after animals are abruptly transferred to a set of high temperatures for a given period of time (e.g., 24 hours) and LT50 is interpolated from the survival curve; and<br>iii) The death time or (or heat knockdown time) where animals are abruptly transferred to elevated temperatures and the time needed for animals to reach an endpoint (e.g., immobilisation, death) is recorded as the response. With the latter measure, the thermal tolerance limit can be inferred from the relationship between the time to death and the temperature of the knockdown assay. Therefore, death times must have been measured at >2 temperatures (e.g., lethal times at 38, 40, and 42°C).<br>We exclude alternative measures of heat tolerance which cannot be converted to the temperature scale (e.g., heat coma recovery time) or CTmax extrapolated from physiological performance curves (e.g., critical temperature for ATPase activity).
4	To be included, the study must have reported the temperature at which animals were maintained in the laboratory (i.e., temperature of acclimation), the temperature of the environment from which animals were captured (i.e., temperature of acclimatization), or the geographical coordinates and dates of capture.

TABLE S5*: Summary of procedural concerns found in some studies.* Note that estimates having procedural concerns were excluded during the data curation (see main text).

**Procedural concerns**	**Number of estimates concerned**
Unclear acclimation conditions	44
Acclimation in outdoor tanks	3
Uncommon or inconsistent thermal tolerance methodology	49
Animals were exposed to high levels to UV radiation	5
Animals were dehydrated prior to testing	3
Animals were starved prior to testing	3
Animals were exposed to predators	1
